# Effect of Almond Supplementation on Non-Esterified Fatty Acid Values and Exercise Performance

**DOI:** 10.3390/nu12030635

**Published:** 2020-02-27

**Authors:** Laura Esquius, Ramon Segura, Guillermo R. Oviedo, Marta Massip-Salcedo, Casimiro Javierre

**Affiliations:** 1 Department of Physiological Sciences, Campus of Medicine and Health Sciences of Bellvitge, Universitat de Barcelona (UB), C. Feixa Llarga, s/n, 08907 Hospitalet de Llobregat, Spain; rasegura@ub.edu (R.S.); cjavierre@ub.edu (C.J.); 2 FoodLab, Faculty of Health Sciences, Universitat Oberta de Catalunya, Avda, del Tibidabo, 39-43, 08035 Barcelona, Spain; mmassips@uoc.edu; 3 Faculty of Psychology, Education and Sport Science-Blanquerna, University Ramon Llull, C. Císter 34, 08022 Barcelona, Spain; guillermorubeno@blanquerna.url.edu

**Keywords:** exercise, performance, almonds, supplementation, non-esterified fatty acids, ergogenic aids, sports nutrition

## Abstract

Several studies have investigated the effects of fat intake before exercise on subsequent substrate oxidation and exercise performance. While some studies have reported that unsaturated fatty acid supplementation slightly increases fat oxidation, the changes have not been reflected in the maximum oxygen uptake or in other performance and physiological parameters. We selected almonds as a fatty acid (FA) source for acute supplementation and investigated their effect on non-esterified fatty acid (NEFA) values and exercise performance. Five physically active male subjects (age 32.9 ± 12.7 years, height 178.5 ± 3.3 cm, and weight 81.3 ± 9.7 kg) were randomly assigned to take an almond or placebo supplement 2 h before participating in two cycling resistance training sessions separated by an interval of 7–10 days. Their performance was evaluated with a maximal incremental test until exhaustion. Blood samples collected before, during, and after testing were biochemically analysed. The results indicated a NEFA value average increase of 0.09 mg·dL^−1^ (95% CI: 0.05–0.14; *p* < 0.001) after active supplement intake and enhanced performance (5389 ± 1795 W vs. placebo 4470 ± 2053 W, *p* = 0.043) after almond supplementation compared to the placebo. The almond supplementation did not cause gastrointestinal disturbances. Our study suggests that acute almond supplementation 2 h before exercise can improve performance in endurance exercise in trained subjects.

## 1. Introduction

In endurance sports, the pattern of energy substrate use changes over time, even when the exercise intensity remains constant. The longer the time spent on exercise, the greater the energy substrate contribution of fat [1,2]. In endurance exercise, the hormone-dependent release and oxidation of plasma fatty acids (FAs) increase in parallel to the gradual exhaustion of muscle glycogen reserves. This increased oxidation occurs in response to increased levels of circulating catecholamine (adrenaline and noradrenaline) and decreased levels of circulating insulin. Catecholamines and insulin play an important role in stimulating and inhibiting lipolytic activity, respectively [3,4].

Trained individuals make greater use of FAs as an energy source and perform better in endurance exercise due to their ability to increase glycogen stores and use them sparingly in submaximal efforts [5,6,7,8]. For this reason, acute FA supplementation has the effect of storing muscle glycogen during prolonged exercise because the oxidation rate of non-esterified fatty acids (NEFAs) partly depends on their blood plasma concentrations [4,8,9].

Previous studies [10,11,12,13,14,15,16,17,18], exploring the effects of acute FA supplementation through high fat intake before exercise, have not demonstrated improved performance. They have, in fact, shown the opposite, with intake causing gastrointestinal problems resulting from delayed gastric emptying induced by long-chain triglycerides (TGs). Those studies have shown that, during endurance exercises, while plasma NEFA availability increases, the pattern of substrate oxidation remains unchanged.

Almonds (*Prunus dulcis*) are highly nutritional, mainly due to their high lipid content (25–66 g per 100 g^−1^ (fresh weight)), which also makes them highly calorific (Table 1). They are very rich in unsaturated FAs, especially oleic acid (monounsaturated) and linoleic acid (polyunsaturated), which account for around 90% of their total lipid content, although proportions vary widely depending on the almond variety. The amounts of saturated FAs they contain, such as myristic, palmitic, and stearic acids, are low (<10%), while the concentrations of carbohydrate, fibre, and protein per 100 g range between 1.8 and 7.4 g, 11 and 14 g, and 14 and 26 g, respectively [19].

Almonds are a good source of α-tocopherol, riboflavin, magnesium, manganese, copper, and phosphorus. They are also rich in arginine, a substrate necessary for nitric oxide [21,22,23]. The phenolic and polyphenolic compounds in almonds include mostly flavonoids, especially isorhamnetin-3-O-rutinoside and catechin [24,25] (Table 2). This nutrient profile has been demonstrated to be important for humans, as the consumption of almonds is associated with improved oxidative stress biomarkers [26,27] and reduced inflammation [28,29] and is inversely related to cardiovascular diseases, diabetes, and certain cancers [26,30,31].

Intense and prolonged physical effort increases reactive oxygen species (ROS) production due to, among other reasons, improved mitochondrial respiration chain oxidation flows; ROS are produced by an electron transfer that requires a high energy input with a very short lifetime (from milliseconds to nanoseconds) [32]. Repeated and programmed exercise improves the ability to defend against ROS. However, ROS overproduction during exercise can overcome antioxidant defence capabilities, causing imbalances in the immune and endocrine systems, inducing fatigue, and impairing performance [33,34,35]. As almonds are a good source of unsaturated FAs, antioxidants, and certain micronutrients, they can help maintain or improve exercise performance by modulating energy use and strengthening antioxidant defences. For example, quercetin [36,37,38,39] may help augment the training effectiveness on exercise performance by up-regulating mitochondrial biogenesis and oxygen sparing capacity and facilitating oxygen delivery to skeletal muscle, and arginine [40,41,42,43,44,45] may decrease ammonia liberation. Nonetheless, the impact of antioxidants and physiological markers on physical performance is not completely known [46].

Based on the assumption that unsaturated FA-rich diets and endurance exercise both have positive (if different) effects on metabolic and cardiovascular health, and given that they both increase the oxidative capacity of fats, their combination is likely to be synergistic [47]. While studies have demonstrated that unsaturated FA supplementation slightly increases fat oxidation after sports training compared to control supplementation, this change has not been reported to be reflected in maximum oxygen uptake (VO_2max_) or other performance and physiological parameters [47,48,49,50].

In our experimental study, we evaluated the effect of acute FA supplementation (almonds), containing unsaturated FA and antioxidant micronutrients, on prolonged resistance training tests in laboratory conditions.

## 2. Materials and Methods

### 2.1. Participants

The participants volunteering in the study were 5 physically active men (age 32.9 ± 12.7 years, height 178.5 ± 3.3 cm, and body mass 81.3 ± 9.7 kg; BMI 24.5 ± 2.2 kg·m^−2^) who perform recreational sports training 3 to 5 days a week. The exclusion criteria were: (1) current or recent injury, (2) intake of fish oil or other FA supplements, and (3) any other condition that could prevent compliance with a maximum exercise test protocol. The study was approved by the institutional ethics committee (Institutional Review Board IRB00003099) and was performed in accordance with the Declaration of Helsinki. Before participating, the subjects read a description of the study and its risks, and signed an informed consent.

### 2.2. Study Design

In this randomised controlled double-blind crossover study, each subject performed the same test procedure on 2 different days (with an interval of 7–10 days) after taking either an active supplement or a placebo supplement. The order of supplementation was randomised using a random number generator (i.e., day 1 = active/day 2 = placebo or day 1 = placebo/day 2 = active). The subjects performed all the tests in a similar postprandial state (3 h after a light meal) and were instructed not to perform intense physical activity in the 72 h before testing.

#### 2.2.1. Supplements

The ingredients of the active supplement were 60 g of ground almonds mixed with 60 mL of milk (to obtain a paste) and 6 g of fructose (as a mild sweetener). The placebo supplement contained the same ingredients (60 mL of milk and 6 g of fructose), but with 100 g of white bread instead of almonds (Table 3). The supplements were prepared in the same laboratory where testing was conducted.

As indicated in Table 3, the active and placebo supplements differed in calorific content by just under 100 kcal, and relatively little in terms of protein levels. The fat content was considerably higher in the active supplement, while the carbohydrate content was lower.

#### 2.2.2. Protocol

To identify the individual workload that corresponded to 50% of the VO_2max_ to be used for submaximal testing, each participant performed a maximal incremental test until exhaustion, with workload increments of 20 W per minute. The test was performed one week before the baseline test (stage 0).

To facilitate multiple blood extractions during the experiments, a venous catheter was inserted into a superficial vein in the forearm of each subject.

**Figure 1:**
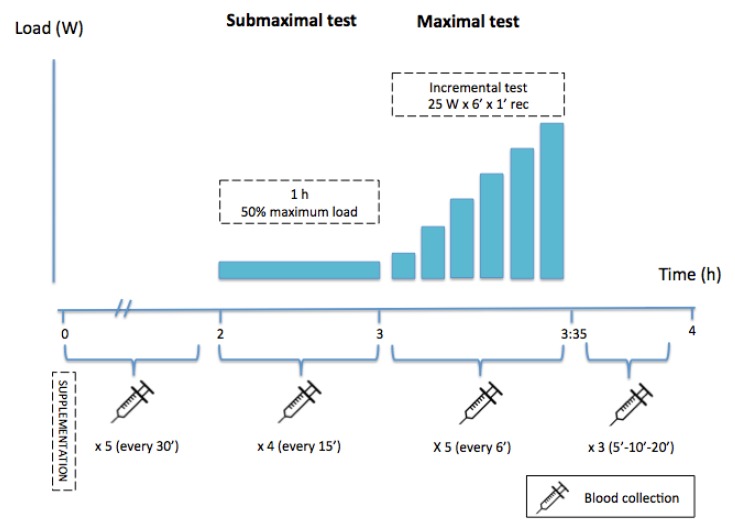
Protocol stages.

The experimental stages (Figure 1) were identical for day 1 and day 2 (active or placebo with the randomised order), as follows:

- Stage 0. Baseline

Subjects took the supplement (active or placebo) and blood was sampled (baseline; minute 0).

- Stage 1. Pre-testing

Blood was sampled at minutes 30, 60, 90, and 120 (every half hour) after supplement intake. The subjects remained inactive during this 2 h period.

- Stage 2. Submaximal test

Subjects performed a 1 h submaximal test at 50% loading (as calculated in the pre-experimental maximal incremental test) and blood was sampled at minutes 15, 30, 45, and 60 (every 15 min).

- Stage 3. Maximal test

With no rest period between Stage 2 and Stage 3, the subjects performed a maximal incremental test involving 6 min steps with power increments of 25 W (starting from individual submaximal workload) followed by 1 min recovery periods until exhaustion. Blood was sampled 5 times in the first part, i.e., at minutes 7, 14, 21, 28, and 35.

- Stage 4. Recovery

After maximal test completion to exhaustion, blood was sampled at minutes 5, 10, and 20 during recovery.

The peripheral venous blood samples were biochemically analysed to determine glucose, lactate, uric acid, urea, cholesterol, TG, NEFA, high-density lipoprotein (HDL), glutamate-oxaloacetate transaminase (GOT), and glutamate-pyruvate transaminase (GPT) concentrations.

### 2.3. Diet

Subjects were instructed not to make changes to their diet during the study period (to avoid changes that might confound the results) and to eat the same light breakfast on each of the test days.

### 2.4. Analytical Procedures

#### 2.4.1. Metabolic Analysis

Subjects performed the tests on pre-calibrated cycle ergometers (Excalibur, Lode, Groningen, The Netherlands). To evaluate differences in the total work performed between the two supplementations, we used the power of each step and the time spent in each workload, assessing the total capacity of the power developed by each subject.

Absolute VO_2_peak (L·min^−1^), relative VO_2_peak (mL·kg^−1^·min^−1^), minute ventilation (VE, L·min^−1^), tidal volume (VT, L), and the respiratory exchange ratio (RER) were measured breath-by-breath using a two-way mask (Hans Rudolph, KS, USA) and an automatic gas analysis system (Metasys TR-plus, Brainware SA, La Valette, France) equipped with a pneumotachometer. The gas and volume calibrations were performed before each test according to the manufacturer’s guidelines.

During the tests, a 12-lead electrocardiogram was performed and the heart rates (HR) of the subjects were continuously monitored (CardioScan v.4.0, DM Software, Stateline, NV, USA).

All tests were conducted during the morning at a room temperature of 22 °C to 24 °C and a relative humidity of between 55% and 65%.

#### 2.4.2. Biochemical Analysis

Glucose, uric acid, urea, cholesterol, TG, HDL, GOT, and GPT concentrations were determined using dry chemistry analysis, performed with the Reflotron^®^ Plus system (Roche Diagnostics, SL, Sant Cugat del Vallès, Spain). Blood lactate was measured using a photocolorimetry system (Vario Photometer, Diaglobal GmbH, Berlin, Germany). NEFAs were analysed using a gas chromatograph (Hewlett Packard, HP6890, USA) equipped with a flame ionization detector; peaks were identified based on the retention time in relation to FAME standards (Supelco, Bellefonte, PA, USA), and peak areas were automatically computed.

### 2.5. Statistical Analysis

Descriptive statistics were calculated for all the variables. To test the normality of the variables, the Shapiro–Wilk test was used. An analysis of variance (ANOVA) for repeated measures was performed to evaluate differences between two different supplements in the blood samples. Student’s *t*-test for paired samples was used to explore differences between the means for the variables measured on test days 1 and 2. Statistical significance was set to *p* < 0.05. Analyses were performed using SPSS 19.0 (IBM SPSS Statistics, Chicago, IL, USA).

## 3. Results

### 3.1. Submaximal Test

The loadings for the submaximal test are shown in Table 4.

The metabolic results for the submaximal test at 50% loading are summarised in Table 5. Following active (almond) supplementation, VCO_2_, RER, and HR values increased significantly (*p* < 0.05) compared to the placebo, but not VO_2_ values.

### 3.2. Maximal Test

The performance results for the maximal test (maximum loads) are summarised in Table 6. An improvement was evident in all five subjects. Performance, overall, improved by 919 ± 705 W (range: 214–1995 W; *t* = 2.91, *p* = 0.043, statistical power = 0.596), reflecting a generalised improvement of 20.6%.

Cardiorespiratory and metabolic data from the maximal tests are summarised in Table 7. Following active supplementation, significant increases were observed in RER, VE and HR (p < 0.05) compared to the placebo, but not in VO2, VCO2, VO2kg, or VT.

### 3.3. Biochemical Analysis

The biochemical data recorded at the end of the submaximal test are summarised in Table 8, demonstrating that TG and NEFA values were higher for the active supplement than for the placebo.

**Figure 2:**
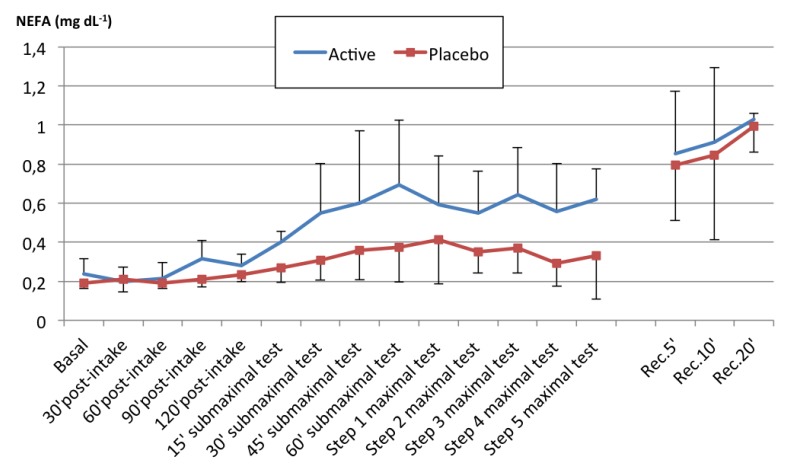
Evolution in NEFA plasma values after active and placebo supplementation. Results are the mean ± standard deviation (SD). A statistically significant increase in the mean values following active supplementation was found (0.09 mg· dL^−1^; *p* < 0.001). Abbreviations: NEFA = non-esterified fatty acids.

After active supplementation, the NEFA values remained higher compared to the placebo, with an average increase of 0.09 mg dL^−1^ (95% CI: 0.05–0.14; *p* < 0.001). Figure 2 depicts the changes in the NEFA values in both cases.

## 4. Discussion

We evaluated the impact of acute FA supplementation in the form of almonds on plasma NEFA values and endurance performance, comparing it to a placebo. Our results indicate that acute almond supplementation modified the energy substrate availability pattern in plasma. In contrast to the little increase produced by the placebo supplementation, almond supplementation before exercise significantly increased plasma NEFA concentrations during the physical test by a mean value of 0.09 mg dL^−1^ (>30% vs. the placebo).

NEFAs, as an oxidisable fuel for physical exercise, can improve performance. According to previous studies [14,16,51,52,53], NEFA availability may save on the use of muscle glycogen during exercise and, thus, delay fatigue. Those previous studies administered heparin to increase plasma NEFA levels; however, while this reduces the oxidation of muscle glycogen, it is not an acceptable pre-competition strategy. Our study demonstrated that NEFA values can be increased using exclusively nutritional strategies. Other studies using fat supplementation unaccompanied by heparin administration [10,11,15] have reported increased NEFA values, but no improvement in performance. By contrast, we found that exercise performance improved by 20.6% following fat supplementation.

An important factor in fat supplementation during exercise is the type of FAs administered. It is important to bear in mind that fat digestion and absorption is a lengthy process, depending on the length of the FA chain, with long-chain TGs, for instance, being absorbed more slowly than short- or medium-chain TGs [54]. Diet almond consumption (>42.5 g) may reduce the risk of CVD by improving blood lipids and by decreasing body weight and apolipoprotein B, but triglycerides, systolic blood pressure, apolipoprotein A1, high-sensitivity C-reactive protein, and lipoprotein (a) showed no difference [55]. In a dose–response study, the results indicated that almond consumption increases oleic acid and monounsaturated fat content in serum triacylglycerol and non-esterified fatty acids fractions, which are inversely associated with CHD lipid risk factors and overall estimated 10-year CHD risk [56]. However, after an acute intake of 60 g of almonds, triglycerides in plasma may be elevated because they are highly nutritional, mainly due to their high lipid content (25–66 g per 100 g^−1^ (fresh weight)), and richness in unsaturated FAs, especially oleic acid (monounsaturated) and linoleic acid (polyunsaturated). In our data, triglycerides increased 14.7% with almond supplementation with respect to placebo after submaximal exercise, with similar dietetic and exercise conditions.

Several studies have evaluated the effects of supplements made with healthy foods rich in monounsaturated FAs, such as almonds, pistachios, and extra-virgin olive oil. In a study by Nieman et al. [57], performance (measured as exercise time) worsened, possibly due to increases in plasma levels of compounds such as raffinose, sucrose, or myo-inositol, accompanied by an increase in leukotoxin (derived from linoleic acid), which may have had a negative impact on mitochondrial function. In studies by Boss et al. [47], Capó et al. [48,49], and Esquius et al. [50], performance (again measured as exercise time) was not affected by supplementation. However, Yi et al. [58], in their evaluation involving 75 g of almonds administered as single pre-exercise supplements over 4 weeks, reported improved performance (measured as distance travelled). While the amount of almonds administered in that study (70 g) was similar to the 60 g administered in our study, the administration protocol differed.

The supplements used in our study were well received by the subjects and did not cause any gastrointestinal problems. The high fat content of the supplements had no negative influence on exercise performance in our study, unlike in other studies reporting impaired physical performance due to fat intake [59]. Digestibility was good and plasma NEFA levels were observed to increase around 90 min after almond supplementation (Figure 2). The increase in plasma NEFA levels occurred at an earlier stage in our study than in other studies [60,61].

The calorie difference between the active supplement (405 kcal) and the placebo (315 kcal) was 90 kcal, while the carbohydrate content was lower. The main difference was in the amount of fat. Further research is warranted to explore differences between different FAs used for acute supplementation in endurance exercise.

Studies in humans have shown that the consumption of almonds increases circulating levels of α-tocopherol in a dose-dependent manner [62,63] and reduces oxidative stress biomarker levels [23,26,27]. The phenolic compounds in almonds have been shown to exert an antioxidant effect against free radicals [27,64] and to decrease inflammatory markers [28,29]. The phenolic and polyphenolic compounds in almonds may, therefore, contribute to improving the antioxidant capacity of athletes (not determined in this study).

No differences were observed for oxygen consumption or CO_2_ production during the submaximal effort, which could have been because there were no differences in the energy substrate used. During the maximal effort, there were also no differences in the maximum oxygen consumption reached, with more CO_2_ being produced. This could indicate that the positive effect on performance was due to the peripheral effect of the polyphenols rather than the energy savings from the extra lipid supplement, resulting in a reduced perception of fatigue that enabled the physical effort to last longer.

The main limitation of this study was the small number of participants and the fact that our sample was composed only of men. The complexity of the protocol, however, needed a homogeneous and trained sample to rule out any training effect. Studies with larger samples would be necessary to confirm our findings, explore individual differences in responses, and test differences arising from other factors such as gender. Since oxidative stress and inflammatory biomarkers could not be assessed in this research, we cannot evaluate the role of phenolic and polyphenolic compounds in almonds in improving the antioxidant capacity of the athletes. Therefore, further research is warranted analysing inflammatory and oxidation markers to confirm this hypothesis.

Our results, in summary, suggest that almonds can be included in pre-training or pre-competition supplements for endurance athletes.

## 5. Conclusions

The acute supplementation with almonds (60 g), administered 2 h before exercise, increases mean plasma NEFA values by 30% and improves exercise performance by 20.6%. Our study suggests that almond supplements could have a positive effect on performance in endurance exercise. Further studies are required to confirm the effects on long-term exercises, using it directly in field tests with larger samples and other population groups (e.g., both sexes).

## Figures and Tables

**Table 1:** Specific nutritional information for a “Marcona almond” (data from Survey (FNDDS), 2019) [20].

Component	Amount	Unit
Water	4.41	g
Energy	579	kcal
Protein	21.15	g
Total lipid (fat)	49.93	g
Fatty acids, total saturated	3.802	g
12:0	0	g
14:0	0.003	g
16:0	3.083	g
18:0	0.704	g
Fatty acids, total monounsaturated	31.551	g
16:1	0.239	g
18:1	31.294	g
20:1	0.005	g
Fatty acids, total polyunsaturated	12.329	g
18:2	12.324	g
18:3	0.003	g
Carbohydrate, by difference	21.55	g
Fibre, total dietary	12.5	g
Sugars, total including NLEA	4.35	g

**Table 2:** Polyphenol composition of the almond (data from Bolling, 2017) [24].

Polyphenol Class	Mean (range 25–75% percentile) (mg/100 g)
Proanthocyanidins (dimers and larger)	162 (67.1–257)
Hydrolysable tannins	82.1 (72.9–91.5)
Flavonoids, non-isoflavones	61.2 (13.0–93.8)
Phenolic acids and aldehydes	5.5 (5.16–12.2)
Minor phenolic constituents (isoflavones, stilbenes, lignans)	0.7 (0.5–0.9)
Sum of classes	312 (161–450)

**Table 3:** Ingredients and nutritional profile of the supplements.

	Active	Placebo
Ingredients	60 g almonds	100 g white bread
	60 mL milk	60 mL milk
	6 g fructose	6 g fructose
Energy (kcal)	405	315
Fats (g)	33.5	1.9
Carbohydrates (g)	12.5	63.2
Proteins (g)	13.5	11.2

**Table 4:** Submaximal test loadings.

Subject	Power (W)	rpm
1	75	60
2	125	60
3	125	60
4	125	70
5	125	70

Abbreviations: rpm = revolutions per minute.

**Table 5:** Submaximal test metabolic results.

	Active		Placebo		
Variable	Mean	SD	Mean	SD	*p*
VO_2_ (L·min^−1^)	2.27	0.25	2.24	0.41	0.085
VCO_2_ (L·min^−1^)	2.12	0.26	2.03	0.35	0.050
RER	0.93	0.00	0.91	0.06	0.000
HR (bpm)	123.00	16.42	120.52	12.15	0.010

Results are the mean ± standard deviation (SD). Abbreviations: VO_2_ = oxygen consumption; VCO_2_ = carbon dioxide production; RER = respiratory exchange ratio; HR = heart rate.

**Table 6:** Maximal test performance.

		Active				Placebo	
Subject	Time in Last Workload (min)	Maximum Power (W)	Total Work (W)	ΔTotal Work (W)	Time in Last Workload (min)	Maximum Power (W)	Total Work (W)
1	3.6	200	4020	1995	4.5	150	2025
2	6	200	3150	663	4	200	2487
3	6	250	6000	500	4	250	5500
4	3.1	250	6134	214	2.5	250	5920
5	2	275	7642	1,225	4	250	6417
Mean			5389	919			4470

**Table 7:** Maximal test metabolic results.

	Active		Placebo		
Variable	Mean	SD	Mean	SD	*p*
VO_2_ (L·min^−1^)	3.38	0.59	3.44	0.52	0.814
VCO_2_ (L·min^−1^)	3.34	0.79	2.88	1.36	0.065
RER	1.00	0.07	0.97	0.08	0.005
VE (L·min^−1^)	100.80	36.3	89.3	31.50	0.000
VT (L)	2.64	0.48	2.90	0.61	0.063
VO_2_kg (mL·kg·min^−1^)	44.44	8.70	45.20	6.90	0.855
HR (bpm)	160.90	24.4	157.5	23.30	0.013

Results are the mean ± standard deviation (SD) in the last workload achieved for each subject. Abbreviations: VO_2_ = oxygen consumption; VCO_2_ = carbon dioxide production; RER = respiratory exchange ratio; VE = ventilation; VT = tidal volume; HR = heart rate.

**Table 8:** Biochemical data after the submaximal test.

	Active		Placebo		
Variable	Mean	SD	Mean	SD	*p*
Lactate (m mol L^−1^)	2.75	2.00	2.86	1.49	0.898
TG (m mol L^−1^)	127.48	46.86	111.19	29.79	0.016
Cholesterol (mg·dL^−1^)	177.29	51.72	182.71	40.72	0.822
Glucose (mg·dL^−1^)	89.09	17.97	89.04	16.27	0.995
Uric acid (mg·dL^−1^)	5.68	0.94	5.91	0.97	0.648
Urea (mg·dL^−1^)	41.02	5.79	40.71	6.32	0.921
HDL (mg·dL^−1^)	34.40	8.09	40.38	11.03	0.001
GOT (UI·L^−1^)	19.23	5.86	33.68	30.69	0.227
GPT (UI·L^−1^)	13.13	7.82	18.10	6.97	0.216

Results are the mean ± standard deviation (SD). Abbreviations: TG = triglycerides; HDL = high-density lipoprotein; GOT = glutamate-oxaloacetate-transaminase; GPT = glutamate-pyruvate transaminase.
